# Mapping the UK renal psychosocial workforce: the first comprehensive workforce survey

**DOI:** 10.1186/s12882-019-1287-0

**Published:** 2019-03-21

**Authors:** Maaike L. Seekles, Paula Ormandy, Emma Coyne

**Affiliations:** 1 School of Health and Society, University of Salford, Room L530, Allerton Building, Salford, M6 6PU UK; 2 School of Health and Society, University of Salford, Room 2.78, Mary Seacole Building, Salford, M6 6PU UK; 3 Renal Unit, City Hospital Campus, Nottingham University Hospitals NHS Trust Hucknall, Road Nottingham, Nottingham, NG5 1PB UK

**Keywords:** Psychosocial, MDT, Workforce, Nephrology

## Abstract

**Background:**

Emerging evidence of psychosocial problems in CKD patients has led to an acceptance that a focus on the emotional wellbeing of the patient should be included in the provision of comprehensive CKD care. It is unclear if an increased attention for psychosocial needs in guidelines and policy documents has led to a rise in psychosocial staffing levels or change in composition of staff since the last workforce mapping in 2002. This paper offers a critical analysis and in-depth discussion of findings and their implications, in addition to providing an international perspective and exposing gaps in current knowledge.

**Methods:**

Data on psychosocial staffing levels was taken from a survey based on the Scottish Renal Association’s (SRA) staffing survey that was sent to all units in England, Wales and Northern-Ireland in 2016. In addition, data from a psychosocial staffing survey designed by and distributed via psychosocial professional groups was used. This data was then completed with Freedom of Information (FOI) requests and collated to describe the current renal psychosocial workforce in all 84 UK renal units. This was compared to results from the last renal workforce mapping in 2002.

**Results:**

The results from this mapping show great variability in models of service provision, significant exceeding of benchmarks for staffing levels, and a change in staffing patterns over the past 15 years. Adult psychology services have increased in number, but provision remains low due to increased patient numbers, whereas adult social work and paediatric services have decreased.

**Conclusion:**

A lack in the provision of renal psychosocial services has been identified, together with the absence of a general service provision model. These findings provide a valuable benchmark for units, a context from which to review and monitor provision alongside patient need. Along with recommendations, this paper forms a foundation for future research and workforce planning. Research into best practice models of service provision and the psychosocial needs of CKD patients lies at the heart of the answers to many identified questions.

**Electronic supplementary material:**

The online version of this article (10.1186/s12882-019-1287-0) contains supplementary material, which is available to authorized users.

## Background

Chronic Kidney Disease (CKD) is a worldwide public health problem, with increasing incidence and prevalence, high costs, and poor outcomes [1]. Forecasted growth in the prevalence of the disease together with predicted further reductions in the nephrology workforce requires forward planning to ensure appropriate management and access to services [2, 3]. Indeed, in the UK too, the population is ageing and with it, the prevalence of CKD and its impact on the health systems grows. It is expected that 2.6 million people (6.1%) aged 16 and older in England have CKD stage 3–5 [4]. According to the 2017 UK Renal Registry report, 61,256 adults received Renal Replacement Therapy (RRT), of which 28,632 dialysis, on the 31st of December 2015 [5]. In addition, 941 children (< 18 years) with established renal failure (ERF) were receiving treatment at paediatric nephrology centres in 2015 [6]. The number of people receiving RRT has grown with 3.9% between 2014 and 2015 [5] and with around 50% over the past decade [7]. CKD, and particularly RRT, poses a high burden on the NHS health care budget. Estimates suggest more than half of the total expenditure on CKD is for RRT, although the RRT population only comprises 2% of the total diagnosed CKD population [7]. For that reason, an important aim of CKD care is to effectively delay or prevent progression of the decrease in renal function and, as a result, the need for dialysis as RRT [8].

Living with CKD provides many ongoing physical, emotional, financial and/or social challenges throughout an individual’s renal journey. Rapidly developing research has shown that these could result in psychosocial problems, not only for patients, but also for families and carers. The most frequently reported psychological disorders in CKD patients are depression, anxiety, and adjustment disorders [9–11]. Recent studies suggest a prevalence of interview-defined depression of approximately 20% in CKD patients [12], with 40% of dialysis patients showing depressive symptoms [13]. In CKD patients, depressive symptoms were found to be independent predictors of adverse clinical outcomes, including faster progression to end-stage-renal disease and thus RRT, increased hospitalization, and mortality [14, 15].

As a result of the physical and psychosocial consequences of the disease and associated comorbidities, the care for CKD patients is complex, multifaceted and often fragmented among different specialties [16]. It is suggested that this type of care is best provided according to a multidisciplinary care model. CKD patients who participate in renal multidisciplinary care which includes psychosocial support show slower renal function decline in advanced stage CKD and improved clinical outcomes, timing initiation of dialysis with functional vascular access and reduced mortality [8, 17, 18].

Even though evidence on psychosocial problems in CKD is still emerging, there is an acceptance that a focus on the emotional and psychosocial needs of the patient should be included in the provision of comprehensive medical care to the CKD patient [11]. It is unclear if this increased attention for psychosocial needs has led to an increase in psychosocial staffing levels or change in composition of staff. Over the past 10 years, several national guidelines and policy documents have highlighted the psychological and social aspects of CKD. These include The National Service Framework for Renal Disease [19, 20]; the current NHS England service specifications [21, 22] and the previous 2014 National Institute for Health and Clinical Excellence (NICE) Chronic Kidney Disease quality standard [23]. However, the revised 2017 NICE quality standards [24] no longer prioritise access to psychosocial support for people with renal disease, why this changed is unclear.

In 2002, the British Renal Society (BRS) executed the last audit of the entire nephrology workforce to date. A workforce plan published the findings from this survey, together with recommendations for establishments and staffing levels across each professional group involved in renal healthcare [25]. For psychosocial care, recommendations were only given for social work and psychology. For RRT patients, a benchmark ratio of 1 Whole Time Equivalent (WTE) social work per 140 RRT patients was advised. The desired psychology ratio was said to be depending on the social work ratio. Namely, if the social work benchmark was met, then the recommended psychology ratio was 1 WTE per 1000 RRT patients. If the social work benchmark was exceeded, a psychology provision of 1 WTE per 500 RRT patients was recommended. In addition, the report offered a recommendation for the dialysis patient-to-social worker ratio, namely 1 WTE per 70 dialysis patients [25].There is limited evidence available to support these recommendations [2]. The report further showed variability in the availability of the recommended types of professionals between units, with few having a full staff complement. Notably lacking were social workers, psychologists and counsellors [26]. Even though the data on psychosocial staff was limited, in adult services, the majority of psychosocial staff was social workers (76%), then counsellors/psychotherapists (16.6%) and then psychologists (7.3%) [25].

This paper presents the key findings of the most comprehensive investigation into the UK psychosocial workforce in 15 years. The aim was to assess the levels of psychosocial staff and map it against the 2002 recommendations and to explore whether there has been a change in size and composition of the current renal psychosocial workforce in the last 15 years. Renal psychosocial services were defined as psychological and/or social care provided by psychosocial staff to meet patients’ informational and emotional needs. Whilst, to a certain extent, nurses might provide this type of care also, the focus of this investigation was only on staff specifically recruited to provide psychosocial support to renal patients. The crude results of this investigation can also be found in a lay report published in 2018 [27]. This paper provides a different presentation and more critical analysis of a selection of the data, offering a more in-depth discussion of findings and their implications. It offers an international perspective, serving as a platform to stimulate other countries to compare and contrast psychosocial service provision. Moreover, it exposes gaps in our current knowledge and, along with recommendations, it forms a foundation for future research and workforce planning.

## Methods

The data that was used to complete this workforce mapping was collected through separate initiatives by a number of collaborating organisations. First, in 2016, the BRS asked Clinical Directors (CDs) of all parent units in England, Wales and Northern-Ireland to complete an excel file, based on the Scottish Renal Association’s (SRA) staffing survey [28]. This workforce survey has been used by the SRA for several years and asks for details on all renal staffing and facilities in the unit, including psychosocial staff. Despite numerous reminders, the response rate to the questions about psychosocial staff was only 64.8%. Coinciding with the BRS survey, an online questionnaire was designed jointly by the British Psychological Society Renal Network, the Renal Psychological Services Group and the British Association of Social Workers Renal Special Interest Group. The link to this questionnaire was distributed through the above networks late 2016 and was envisaged to be completed by all individual members of renal psychosocial staff in the UK (including Scotland). Psychosocial staff were asked to provide information on a wide range of questions about qualifications, WTE, type of interventions provided, time in post, funding of post and concerns about the service, among others. Since the overall number of psychosocial staff members was not known, response rates were hard to define. However, 104 professionals returned the questionnaire, which, based on current findings, would mean a response rate of 55.3%. In 2017, Kidney Care UK (KCUK), in collaboration with the University of Salford, supported a researcher to combine, clean and validate the results of both surveys to provide an overview of the psychosocial staffing levels per unit. Conflicting information was found, with renal psychosocial staff contradicting information provided in the BRS workforce survey. Therefore, Freedom of Information (FOI) requests were sent to all 84 hospitals, asking for details only about the type, number and WTE of renal staff. The response rate was high, with 96.4% of units replying to this request within the end date of the data collection period. In addition, email contact between the researcher and renal staff took place to ask for clarification of data. Finally, 100% of the units provided information through at least one of the data collection methods. Whilst data triangulation increased the reliability of the data, all CDs were then asked to confirm accuracy of the findings with 82% of CDs adhering to this request.

The data was managed in Excel and a selection of data - number and WTE of staff – were analysed using Stata 14 software for summary and descriptive statistics to investigate the research questions of this publication. To calculate staff-to-patient ratios, the adult CKD, RRT and paediatric ERF patient number data was obtained from the 19th UK Renal Registry Report [5, 6]. Every attempt was made to collect complete data in the current investigation, however, there was some missing data, with not all WTEs provided for all staff. This had consequences for the calculations of totals and ratios. Namely, the total WTE per profession with more than 10 staff identified was calculated based on assigning the average WTE to the missing data. This was done for three out of 68 social workers and three out of 64 psychologists. Staff-to-patient ratios were only calculated for the units that employ psychosocial staff and provided information on WTEs of staff. Information on the number of young adult patients (aged 16 to 24) per unit was not available and therefore individual youth worker ratios could not be calculated.

Data as presented in the 2002 workforce report was used to compare the current findings to those 15 years ago and assess current ratios against the set benchmarks. The 2002 report only provided information of WTE of social workers and psychologists and did not provide information on variation in ratios or whether benchmarks had been met, which limited the possibilities for comparisons. In addition, it only provided benchmarks for social work and psychology, and not for other psychosocial staff.

## Results

### 2017 Establishment of renal psychosocial workforce

Table 1 shows the identified staffing levels in adult and paediatric services as per July 2017 across 84 renal units in the UK, a complete list of identified psychosocial staffing per parent unit can be found in Additional file 1. It was observed that some of the units employed non-traditional members of psychosocial staff, such as a cultural and health liaison officer. Three units contracted external companies, namely Auriga and Citizens Advice Bureau (CAB), to provide patients with welfare and benefits advice. The four main providers of psychosocial services in the nephrology setting were identified as social workers (35.6, 95% confidence interval [CI] =28.8, 42.8), psychologists (34%, CI = 27.3, 41.2), counsellors/psychotherapists (15.7%, CI = 10.5, 20.7) and youth workers (5.2%, CI = 2.1, 8.4). Of these four types of professionals, 12.4% worked in paediatric services, whereas only 1.5% of the entire RRT population is under 18 years of age. The numbers of psychosocial staff available to renal patients in a unit varied from zero to seven (*Mdn* = 2.37, *IQR* = 1–3.75). Twelve units (14.3%, CI = 7.6, 23.6) had no renal dedicated psychosocial service and sixteen units (19.1%, CI = 11.3, 29.1) had one member of psychosocial staff available to patients. In the other units, different members of staff worked together in varying combinations.

**Table 1:** Number, WTE and proportion of psychosocial staff with 95% confidence intervals

Adult services				Paediatric Services			
Profession	N	WTE	% of total N	Profession	N	WTE	% of total N.
Social work	58	44.6	36.3 [28.8, 44.2]	Social work	10	7.4	32.3 [16.7, 51.4]
Psychology	51	27.2	31.9 [24.7, 39.7]	Psychology	14	5.7	45.2 [27.3, 63.9]
Counselling/ Psychotherapy	28	15.0	17.5 [11.9, 24.3]	Play therapy	2	2.0	6.5 [0.8, 21.4]
Youth work	9	6.5	5.6 [2.6, 10.4]	Play worker (unqualified)	2	2.0	6.5 [0.8, 21.4]
Welfare advisor	3	2.2	1.8 [0.4, 5.4]	Counselling/ psychotherapy	1	0.5	3.2 [0.08, 16.7]
Social care practitioner	1	1.0	0.6 [0.02, 3.4]	Youth work	1	0.25	3.2 [0.08, 16.7]
Assessment & support coordinator	1	0.85	0.6 [0.02, 3.4]	Music therapy	1	0.2	3.2 [0.08, 16.7]
Cultural & health liaison officer	1	0.8	0.6 [0.02, 3.4]	Total	31	17.05	
Trainee CB^a^ therapist	1	0.4	0.6 [0.02, 3.4]				
Psychiatrist	2	Not known	1.3 [0.2, 4.4]				
Psychology assistant	2	Not known	1.3 [0.2, 4.4]				
External companies (Auriga and CAB)	3	Not known	1.8 [0.4, 5.4]				
Total	160	Not known					

^a^CB therapist stands for Cognitive Behavioural Therapist

### Patient-to-staff ratios

Table 2 shows the median and dispersion of adult patients per 1 WTE staff per unit, for psychologists, social workers and counsellors/psychotherapists. Dialysis patients include all dialysis modalities and RRT also includes transplant patients. In addition, the table shows the proportion of units that meet the benchmark ratios as recommended in the 2002 workforce report [25]. There are no units that meet the social work requirements, which means that the psychology requirement for all units is 1 WTE per 500 RRT patients. Only four units meet this requirement, three of which are in Wales. Some units that employ psychologists also offer a counselling service. Taking the counselling and psychology provision together would mean that one additional unit has a provision of 1 WTE psychologist and counsellor for less than 500 RRT patients. It is however not specified in the 2002 recommendations how the presence of counselling services in a unit affect the psychology requirements.

**Table 2:** Ratio of adult patients per 1 WTE staff per renal unit

	No. of units	Mdn	Q1	Q3	IQR	Proportion of units meeting benchmarks 95% CIs
Psychology	33					
Dialysis		675	368	1290	922	No benchmark available
RRT		1392	838	2665	1827	4.7% [1.3, 11.7]
Social Work	32					
Dialysis		311	195	385	190	0
RRT		614	396	929	533	0
Counselling/ Psychotherapy	15					
Dialysis		591	298	905	607	No benchmark available
RRT		1358	905	2035	1130	No benchmark available

**Fig. 1:**
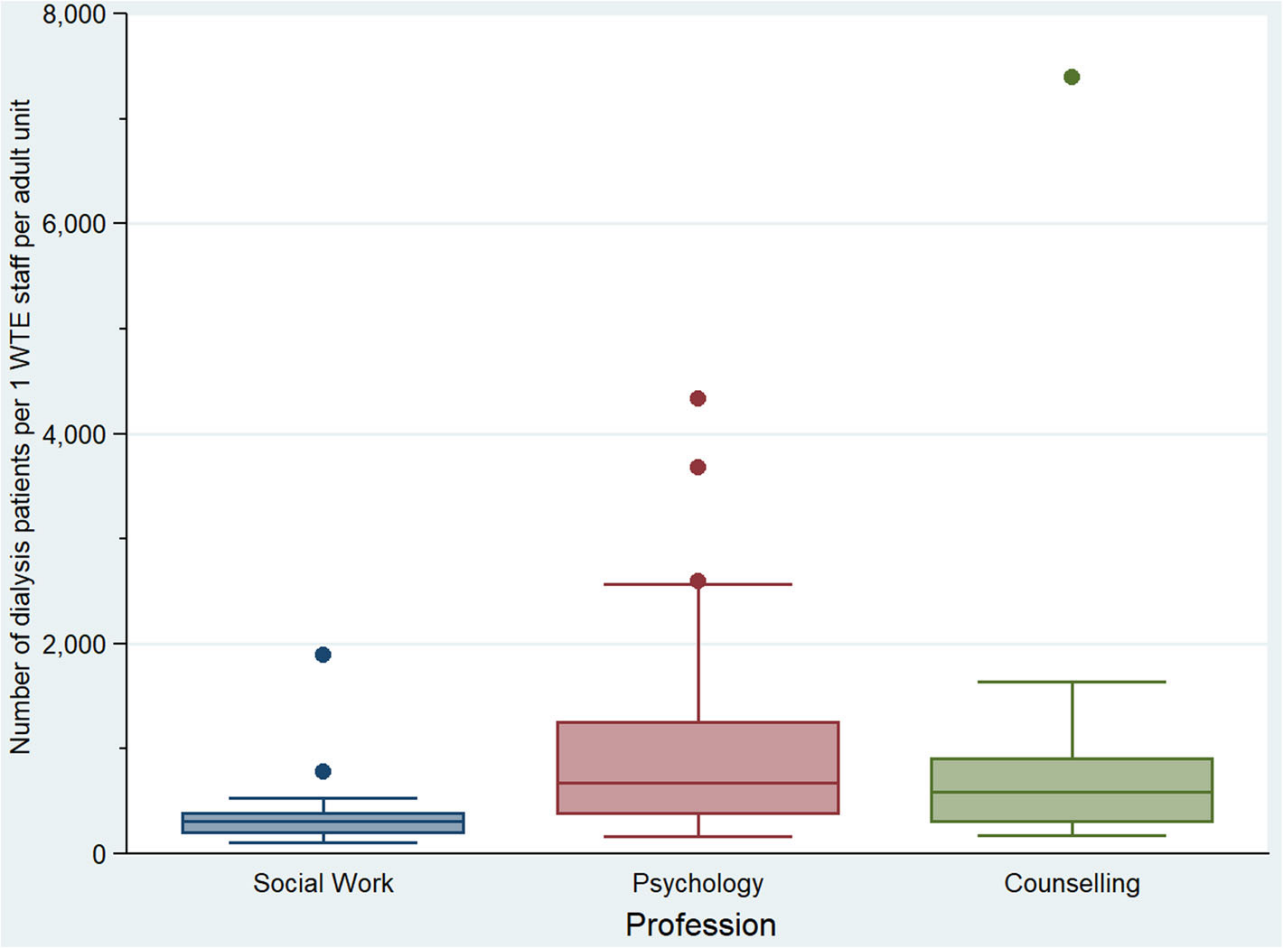
Variation in dialysis patient-to-staff ratio per profession

**Fig. 2:**
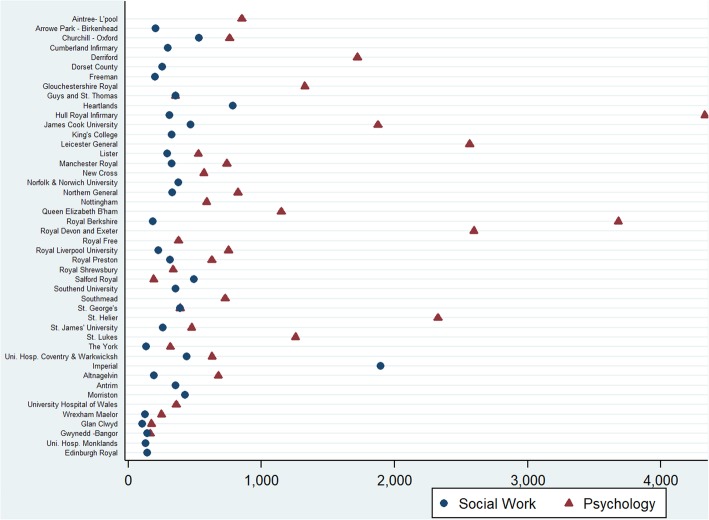
Social work and psychology staff- to- dialysis patient per unit

Notable variations exist between the staff to dialysis patient ratios across different units. For social work, the unit with the best staff ratio per dialysis patient had a ratio of 104, exceeding the benchmark with 48%. This was 165 for psychology and 171 for counselling. The worst ratio for social work was 1895. This was 4430 for psychology and 7390 for counselling. Figure 1 shows the variation in dialysis patient-to-staff ratios per profession. Ratios for RRT patients showed similar variations. Figure 2 provides an overview of the different ratios across all units that have psychology and/or social work provision.

Differences in median ratios for adult psychology and social work services across the four UK countries have been observed (Table 3). No renal counsellors/psychotherapists were identified in Wales and Scotland, in Northern-Ireland one unit was found to employ a counsellor. Most renal units in Scotland (5 out of 9) do not have any renal dedicated psychosocial staff, but instead operate a general model of psychosocial service provision. Ratios for psychology and social work services were the lowest in Wales.

**Table 3:** Ratios of adult dialysis patients per 1 WTE staff per country

	No. of units with provision	Mdn	Q1	Q3	IQR	% of adult units offering psychosocial services
England						
Social work	24	328	257	414	157	90.3
Psychology	28	745	500	1522	1022	
Wales						
Social work	4	132	114	284	170	80.0
Psychology	4	211	169	304	135	
Northern-Ireland						
Social work	3	274	193	355	162	80.0
Psychology	1	675	–	–	–	
Scotland						
Social work	2	137	132	141	9	33.3
Psychology	0	–	–	–	–	

The ratios for number of ERF patients per 1 WTE staff in paediatric services are summarized in Table 4. The ratios in paediatric services were substantially lower than in adult services, which means that even though there are far fewer children with ERF than adults on RRT, relatively more staff is employed in paediatric units. The 2002 recommendations for paediatric services were based on WTE staff per million population of the entire region that a hospital served [25] and not on the number of renal patients in paediatric services. For that reason, it could not be usefully determined whether the found ratios met the set requirements.

**Table 4:** Median and quartiles numbers of ERF paediatric patients per 1 WTE staff

	No. of units	Mdn	Q1	Q3	IQR
Social work	8	71	41	105	64
Psychology	11	93	71	220	149
Play therapy	2	60	32	87	55

### Comparisons to 2002 workforce

As previously stated, the 2002 workforce report only provided complete information on workforce data for renal social workers and psychologists. Therefore, changes in the workforce since 2002 can be reported for renal psychologists and social workers only. The total WTE of renal psychologists has increased with 1088%, from 2.5 to 27.2 WTE. Instead, the adult social worker WTE has decreased with 19%, from 55 to 44.6 WTE. The percentage of adult units without social worker input has increased with 252.1%, from 14 to 49.3%. In addition, the results show that in 2017, 29.6% of units had no psychology or social work support available. Only four units (4.7%, CI = 1.3, 11.7) provided adequate psychology provision and for all units the social work provision was inadequate. The 2002 workforce report did not offer any data on adequacy of provision to compare against.

Taken together, the adult workforce of these psychosocial professions has increased with almost 25% over the past 15 years. However, over the same time, the RRT population has increased with over 50%. Table 5 shows a comparison of overall adult staff to patient ratios, including units without psychosocial services.

**Table 5:** Average 1 WTE adult staff- to- patient ratios for all units

	2001		2017		2002 Recommendations	
	*Dialysis*	*RRT*	*Dialysis*	*RRT*	*Dialysis*	*RRT*
Renal Psychologists^a^	Unavailable	1: 15233	1: 1053	1: 2252	Not known	1: 1000/500
Renal Social Workers	Unavailable	1: 693	1: 642	1: 1373	1: 70	1: 140

^a^When taking psychology and counselling/psychotherapy services together, the 2017 establishment is 1:668 for dialysis patients and 1:1429 for RRT patients

Paediatric renal psychology and social work services have decreased with 20.6% over the last 15 years. Psychology provision decreased with 6.6%, from 6.1 to 5.7 WTE and social work services decreased with 28.9% from 10.4 to 7.4 WTE. In 2002, it was reported that three paediatric units (21.4%) did not have social work input and six units (43%) had no psychology input. Now, the number of paediatric units without social work input has increased with 66.7%, to five units (35.7%). Instead, the number of units without psychology input has decreased with 66.7%, to two units (14.2%). All paediatric units have at least one social worker or psychologist present, even though in one of the units the psychosocial service is made up of a psychologist who works less than 0.1 WTE on renal services.

## Discussion

This investigation set out to assess the current renal psychosocial workforce, to map it against 2002 recommendations and compare it with staffing levels found in 2001. Limitations to this audit included incomplete and conflicting provision of data by the renal units, because of which the total WTE of staff for psychologists and social workers had to be calculated based on average numbers, and the ratios could not be calculated for all units. Uncertainties about the accuracy of the 2002 workforce report complicated the comparison of 2001 data to 2017 data. However, every attempt has been made to extrapolate an accurate and reliable workforce data set, overcoming inconsistencies and non-responses for data items. The researchers achieved a 100% response rate from listed renal units and 82% of units confirmed that their data was correct. Given this, the reported data can be considered highly accurate.

### Variation in renal psychosocial staffing

The results show a great variety in models and availability of psychosocial services within the 84 renal units and UK countries, with Wales reporting the best staff-to-patient ratios. These findings suggest that a general service provision model for renal psychosocial care in the UK is lacking. Guidelines that state patients must ‘*have access to’* psychosocial services [21–23] hardly seem strict or specific enough. This suggests renal units do not have to employ staff to provide these services, nor do they clarify the type and number of staff that should be accessible. At a very pragmatic level, adequacy of staffing ultimately determines whether guidelines issued to improve care and safety are implemented [29]. However, sufficient consideration of staffing levels and how they may be a factor in suboptimal care seems to have been overlooked when drafting these documents.

### Psychosocial staffing models

The recommendations as set out in the 2002 report provided guidance for the provision of social work and psychology. An update of these recommendations should be considered, since it is currently unclear how the presence of counselling and youth services in a unit affects the social work and psychology requirements. Moreover, a recommendation for paediatric services based on the number of children with ERF is lacking. Even though the recommendations for social work are similar to those in Australia and the USA, the evidence base for these benchmarks has been questioned [2, 29]. To inform the development of future recommendations, it is useful to explore and compare recommended levels of provision in other physical health specialities in which psychosocial workforce planning has evolved. In paediatric and adult cystic fibrosis (CF), the care standards require 1 WTE psychologist and 1 WTE social worker per 150 patients [30]. This model involves providing all patients with routine psychosocial care and not stratifying on the basis of psychosocial need. The recommended levels for social work provision are broadly similar in both CF and RRT, but the recommendations for psychology services differ quite considerably. These seem to be more in line with those in cancer care, where NICE estimates that 15% of patients will need level 3 (counsellor/psychologist) support and 10% will need level 4 (psychologist/psychiatrist) intervention [31]. This has led to the development and application of a cancer care model for psychological provision across London [32], which suggests a maximum yearly caseload of 150 patients per full-time level 3/4 worker. It should be noted that this oncology model is for psychological intervention only and not social work services. In 2014, these services appeared to be mostly provided by charity funded oncology social workers and social services [33]. Regardless of whether it is desirable that the help of charities is needed to provide access to social work, this suggests that the variety in psychosocial service provision is not limited to kidney care, but exists within care pathways across other long-term conditions. Applying the cancer care model to renal care would allow for the inclusion of counsellors/psychotherapists in the recommendations and the use of an acuity model; of every 600 RRT patients, 150 patients would require level 3/4 intervention. Sharing of psychosocial provision across other long-term conditions such as diabetes and oncology, may be a way this could be operationalised for smaller units, indeed in some areas this is already established practice. However, even though patients with chronic conditions seem to experience an impact of their disease in similar psychosocial areas, it cannot be assumed that the need for psychosocial services is the same in both CKD and oncology patients.

### Exceeding benchmarks

The above comparisons suggest that the renal recommended staffing levels from 2002 are in line with a social work provision model of providing support to all RRT patients and a psychological provision model of providing support to approximately 25% of the RRT population. However, these recommendations should also include pre-dialysis and palliative care work [25], which is now not clearly reflected in the recommendations based on RRT patients. The survey findings show that not one of the 84 renal units meet the requirements for psychology and social work services together, with average staff-to-patient ratios far exceeding the benchmarks. Based on the recommendations, the current staffing requirement for adequate adult renal social work would be 437.8 WTE and for psychology services this would then be 61.3 WTE. Given that the social work establishment currently is 44.6 WTE, it should be questioned how realistic these benchmarks are.

Although health systems and training of professionals vary across countries, studies from the USA [29] and Australia [2] show that the exceeding of social work benchmarks appears to be a consistent theme that fits within a wider, global shortage of the whole nephrology workforce [3]. In 2011, American renal social work benchmarks were exceeded in 10 out of 50 states. Apart from one state reporting a ratio of 1:425, the median ratio for other states exceeding the recommendations for dialysis patients was 1:150 [29]. In Australia, a recent study in Queensland showed a ratio of 1:191 dialysis patients, whereas one in Western Australia in 2007 calculated ratios of 1:322 [2]. Even though it should be noted that these countries seem to have less renal psychological provision than the UK, the overall UK renal social worker-to-dialysis patient ratio of 1:624 is considerably higher. Indeed, a lack of UK psychosocial service provision appears to be identified by this investigation. Yet, this cannot be concluded based on the current evidence, or lack thereof. Further research into psychosocial problems and need for services is required, to provide an evidence base to support and update 2002 staffing recommendations.

### A changing workforce

The comparison of the current workforce with the 2001 workforce shows that overall, the WTEs of psychologists and social workers have increased with 12.8%, thanks to the large (1088%) increase of adult psychology staff. Instead, paediatric psychosocial services and adult social work saw their workforce decreasing. The results show an interesting trend, namely that the combined psychology and counselling/psychotherapy workforce has become almost the same size as that of the social workers. Indeed, the increase of psychology services seems to have come at the cost of social work services. The 2002 report shows that traditionally, renal social workers provided psychosocial support for CKD patients, as still seems to be the case in the USA and Australia [2, 29]. The employment of renal psychologists and counsellors/psychotherapists is a relatively new and unique phenomenon in the UK, leading to the creation of new service provision models. Even though this shift could be a result of the increasing evidence on psychological problems and interventions with CKD patients – or possibly the lack of high-quality studies that could form an evidence base for renal social work – the reasons for units deciding to employ certain members of staff requires clearer understanding. However, with benchmarks for social work far exceeded, one can wonder if there is enough time for social workers to provide the complex case work activities that they are trained to do. Indeed, renal social workers in the USA have reported that high caseloads prevent them from providing adequate support and that, in their view, patients are being denied access to good quality services [34]. There is a need to investigate whether this is also the case in the UK, manifesting in an unmet need for support, or that patients access services through other routes if adequate dedicated provision of psychosocial care in their unit is lacking. It is unclear whether in these units the role of the nurses is expanded to include a certain level of psychosocial support, or whether patients access psychosocial services through the general hospital team or their GP. Moreover, the effectiveness of other models of service provision needs to be evaluated, to determine whether there is a difference in patient outcomes of services provided by renal dedicated staff or general staff.

### Recommendations for future workforce audits

As described above, the data collection process for this paper was complicated and time-consuming and it requires improvement to make it appropriate for regular, future psychosocial workforce audits. Namely, the BRS workforce survey (based on the SRA survey) was a large document that would take time to complete, which might explain the low response rates. In addition, if the psychosocial section of this larger survey contained no data, it was unclear whether this was because there was no staff available, or whether the section was not completed. Moreover, the contradictory information found between data from CDs and psychosocial staff could suggest that staff roles are not always clear. Also, the survey asked to list renal dedicated psychosocial staff and thus did not include general psychosocial services that patients might have access to. This might explain the differences in findings from a national survey into renal young adult transition services in 2016, which found a higher number of units having access to psychology or youth work services [35], than found in the current investigation. Intuitively, a renal dedicated psychosocial service is provided by any member of psychosocial staff, with time specifically allocated for renal patients, who is funded through the renal budget or a renal charity. Future workforce audits could include a question around funding streams of services, to increase understanding around different funding models of psychosocial care.

Instead, the questionnaire distributed through the psychosocial professional groups provided detailed information that was likely to be accurate, as it came directly from psychosocial staff. However, since the questionnaire was distributed through the professional networks there was a high possibility that it did not reach all members of psychosocial staff, especially those that are not members of the professional groups. Moreover, this questionnaire would not reach the units that do not have any psychosocial provision, therefore not allowing them to confirm their lack of services. The FOI requests proved useful for collecting data from units that did not initially respond, since hospitals have a legal obligation to reply to FOI requests within 20 working days [36]. The confirmation of final results by CDs was believed to increase the reliability of findings and did not show any indications of response bias. Namely, the CDs that did not confirm results were thought to have varying numbers of psychosocial staff available to their patients.

Through combining the learning from all different data collection methods used in this paper the following recommendation can be made: Future psychosocial workforce audits should use a simple electronic survey, asking about type, number, WTE and funding of psychosocial staff. This survey should be completed by psychosocial staff where available, but should be send to CDs of all units, either directly via email or via FOIs, to warrant a return if there is no psychosocial staff available.

## Conclusion

While there are clear limits to our understanding, this investigation can be seen as a robust baseline from which to explore further research and more regular future workforce audits. The results from this mapping show great variability in models of service provision. It appears that the lack of clear policies and guidelines about the provision of psychosocial services has given units the freedom to design their own models, with some units seemingly prioritising psychosocial care more than others. Moreover, significant exceeding of benchmarks for staffing levels and a change in staffing patterns over the past 15 years have been observed. It is unclear whether the apparent lack of psychosocial services influences the ability of staff to adequately fulfil their tasks and whether it has consequences for the psychosocial wellbeing of patients. Research into the psychosocial needs of CKD patients lies at the heart of the answer to many identified questions. Moreover, there is a need to investigate current models of psychosocial service provision and to identify and share good practice of how best to address the needs of patients. Ultimately, the outcomes of these investigations could guide the development of an evidence-based psychosocial care pathway. Similar to findings 15 years ago, our investigation today shows that renal dedicated psychosocial staff is lacking, suggesting that formal emotional and psychological support is often seen as a relatively low priority, especially in a financially constrained, medically driven environment [26].

## Additional file

Additional file 1: Psychosocial Provision per Renal Unit in July 2017. An overview of data on the availability of renal dedicated psychosocial staff per renal unit as per July 2017. The unit name is shown in bold if the data is confirmed by the clinical director. (DOCX 24 kb)

## Abbreviations

BRS: British Renal Society
CAB: Citizens Advice Bureau
CD: Clinical Director
CF: Cystic Fybrosis
CI: Confidence Interval
CKD: Chronic Kidney Disease
ERF: Established Renal Failure
FOI: Freedom of Information
KCUK: Kidney Care UK
NICE: National Institute of Clinical Excellence
RRT: Renal Replacement Therapy
SRA: Scottish Renal Association
WTE: Whole Time Equivalent
