# Supplementary material for: Mapping the UK renal psychosocial workforce: the first comprehensive workforce survey
Source: BMC Nephrol. 2019 Mar 21;20:100. doi: 10.1186/s12882-019-1287-0 (PMC6427898; doi:10.1186/s12882-019-1287-0)
Supplement: Supplementary file 1 — Psychosocial Provision per Renal Unit in July 2017. An overview of data on the availability of renal dedicated psychosocial staff per renal unit as per July 2017. The unit name is shown in bold if the data is confirmed by the clinical director. (DOCX 24 kb) [file 12882_2019_1287_MOESM1_ESM.docx]

**Additional file 1 -** Psychosocial Provision per Renal Unit in July 2017 *– Confirmed data in bold.*

**Units in England**

| **Name of renal unit** | **Staff available** | **Number of sessions on renal services** |
| --- | --- | --- |
| **Addenbrooks** | 2 counsellor/psychotherapist  1 Social Care Practitioner | 5 + 4 sessions  10 sessions |
| **Aintree University Hospital** | 3 psychologists + 1 assistant | 2.5 sessions |
| **Alder Hey Children’s Hospital** | 1 psychologist | 3 sessions |
| **Arrowe Park Hospital** | 1 social worker | 10 sessions |
| **Barts & The London Hospital** | 1 assistant psychologist  1 psychiatrist  Recruiting 1 psychologist  CAB | 6 sessions  unknown  unknown |
| **Basildon University Hospital** | 1 counsellor/psychotherapist | 4 sessions |
| **Birmingham Children’s Hospital** | 1 psychologist  1 social worker  2 play workers (Not qualified) | 5 sessions  10 sessions  20 sessions |
| **Bristol Royal Hospital for Children** | 1 psychologist  1 social worker | 6 sessions  10 sessions |
| **Broomfield Hospital** | 2 counsellor/psychotherapist | 4 + 6 sessions |
| **Churchill Hospital** | 1 psychologist  1 social worker  1 youth worker | 7 sessions  10 sessions  10 sessions |
| **Colchester General Hospital** | No renal psychosocial staff available | - |
| Cumberland Infirmary | 1 social worker | 4 sessions |
| Derriford Hospital | 1 psychologist  1 Youth/Young adult worker | 1 session  2 sessions |
| **Doncaster Royal Infirmary** | No renal psychosocial staff available. Patients who are also receiving care from Northern General (mostly transplant patients) are seen by the psychosocial team of Northern General. |  |
| **Dorset County Hospital** | 2 social workers  2 counsellor/psychotherapist  1 assessment & support coordinator | 8 + 5 sessions  5 sessions  8.5 sessions |
| **Evelina Children’s Hospital** | 1 social worker  1 music therapist  2 psychologists  Play specialist team | 10 sessions  2 sessions  14 sessions |
| **Freeman Hospital** | 3 social workers  1 youth/young adult worker | 6+6+6 sessions  10 sessions |
| **Glouchestershire Royal Hospital** | 1 psychologist | 2 sessions |
| **Great North Children’s Hospital** | 2 psychologists  1 social worker | 5+7 sessions  10 sessions |
| **Great Ormond Street Hospital** | 1 psychologist  1 counsellor/psychotherapist  2 social workers | 4 sessions  5 sessions  5+5 sessions |
| **Guy’s and St Thomas’s Hospital** | 2 psychologists  2 social workers  1 youth/young adult worker | 10 + 10 sessions  10 + 10 sessions  5 sessions |
| **Heartlands Hospital** | 1 social worker | 6 sessions |
| Hull Royal Infirmary | 1 psychologist  2 social workers | 1 session  10+4 sessions |
| **Ipswich Hospital** | 1 counsellor/psychotherapist | 2 sessions |
| **James Cook University Hospital** | 1 psychologist  1 social worker (on sick leave for the past 6 months) | 2 sessions  8 sessions |
| **Kent & Canterbury Hospital** | 2 counsellors/psychotherapists  1 welfare officer | 6 + 10 sessions  10 sessions |
| **King’s College Hospital** | 2 social workers  3 counsellors/psychotherapists | 10 + 10 sessions  10 +7 + 5 sessions |
| **Leeds Children’s Hospital** | 1 psychologist | 5 sessions |
| **Leicester General Hospital** | 1 psychologist (only main unit) | 4 sessions |
| **Lister Hospital** | 1 psychologist  2 social workers  2 counsellors | 10 sessions  10 +8 sessions  8 + 4 sessions |
| **Manchester Royal Infirmary** | 1 psychologist  2 social workers  1 counsellor | 8 sessions  10+8 sessions  10 sessions |
| New Cross Hospital | 2 psychologists  1 welfare officer | 3 + 4 sessions  2 sessions |
| **Norfolk & Norwich University Hospital** | 1 social worker  1 counsellor | 10 sessions  5 sessions |
| **Northern General Hospital (Sheffield Kidney Institute)** | 2 psychologists  1 Trainee CB therapist  3 Social workers | 4 + 4 sessions  4 sessions  20 sessions |
| **Nottingham Children’s Hospital** | 2 Social workers  1 play therapist | 5 + 5 sessions  10 sessions |
| **Nottingham City Hospital** | 1 psychologist  1 youth worker | 8 sessions  5 sessions |
| Queen Alexandra Hospital | 1 counsellor/psychotherapist | 1 session |
| Queen Elizabeth Hospital | 2 psychologists  2 counsellors/psychotherapists  3 welfare officers (external) | 5 + 5 sessions  3 + 4 sessions  Unknown |
| **Royal Berkshire Hospital** | 1 psychologist  2 social workers | 1 session  10 + 10 sessions |
| Royal Cornwall Hospital | No renal dedicated psychosocial staff |  |
| **Royal Derby Hospital** | 1 Youth/young adult worker | 5 sessions |
| **Royal Devon and Exeter Hospital** | 1 psychologist  CAB welfare advice (funded by PA) | 2 sessions  1 session |
| Royal Free Hospital | 3 psychologists  1 youth/young adult worker | 10 + 8 + 5 sessions  10 sessions |
| **Royal Liverpool University hospital** | 1 psychologist  2 social workers | 6 sessions  10 + 10 sessions |
| Royal Manchester Children’s Hospital | 1 psychologist | <1 session |
| **Royal Preston Hospital** | 1 psychologist  2 social workers (should be full time, but 1 on 12 month maternity leave, 1 post half filled) | 10 sessions  10+10 sessions |
| Royal Shrewsbury Hospital | 1 psychologist | 7 sessions |
| **Royal Sussex county Hospital** | 4 counsellors  1 welfare officer | 6 + 6 + 6 + 5 sessions  10 |
| Russells Hall Hospital | 3 psychologists | unknown |
| **Salford Royal Hospital** | 3 psychologists  1 social worker | 10 + 10 + 6 sessions  10 sessions |
| **Southampton Children’s Hospital** | 1 renal psychologist  Play therapist  Currently aiming to get a youth worker | 4 sessions  10 sessions |
| **Southend University Hospital** | 1 social worker | 4 sessions |
| **Southmead Hospital** | 2 psychologists (1 on maternity leave, not covered)  1 youth worker | 6 + 2 sessions  0.5 session |
| St George’s Hospital | 1 psychologist  1 social worker  1 youth worker | 10 sessions  10 sessions  10 sessions |
| **St Helier Hospital** | 1 psychotherapist  In the process of hiring a social worker | 4 sessions |
| **St James’s University Hospital** | 2 psychologists  3 social workers | 7 + 5 sessions  10 + 6 + 6 sessions |
| **St Luke’s Hospital** | 1 psychologist  1 Cultural and health Liaison officer | 2 sessions  8 sessions |
| **Sunderland Royal Hospital** | No renal psychosocial staff available. Sunderland has a large psychology department but has no specified sessions allocated. Also Psych Liason. | - |
| **The York Hospital** | 2 psychologists  2 social workers | 3 + 3 sessions  7 + 7 sessions |
| **University Hospital Coventry & Warwickshire** | 1 psychologist  2 social workers | 7 sessions  5 + 5 sessions |
| University Hospital of North Staffordshire | No renal psychosocial staff available | - |
| **West London Renal & Transplant Centre (Imperial)** | 1 social worker  2 counsellors/psychotherapists | 8 sessions  8 + 6 sessions |

**Units in Northern Ireland**

| **Name of renal unit** | **Staff available** | **Number of sessions on renal services** |
| --- | --- | --- |
| **Altnagelvin Area Hospital** | 1 psychologist  1 social worker | 2 sessions  7 sessions |
| **Antrim Area Hospital** | 1 counsellor/psychotherapist  1 social worker | 8 sessions  4 sessions |
| Belfast City Hospital | No renal dedicated staff available. Hospital psychologists (3) and social work team. |  |
| Daisy Hill Hospital | 2 social workers | unknown |
| **Royal Belfast Hospital for sick children** | 1 psychologist  1 social worker | 6 sessions  6 sessions |
| **Ulster Hospital** | 1 social worker | As required. The dedicated social worker has been drafted in to ease crisis in unscheduled care |

**Units in Wales**

| **Name of renal unit** | **Staff available** | **Number of sessions on renal services** |
| --- | --- | --- |
| **Children’s Hospital for Wales** | 1 social worker  1 youth/young adult worker (shared) | 8 sessions  2.5 sessions |
| **Morriston Hospital** | 1 social worker  1 youth worker (shared) | 10 sessions  5 sessions |
| **University Hospital of Wales** | 3 psychologists  1 youth worker (same person as at Morriston) | 5 + 5 + 6 sessions  2.5 sessions |
| **Wrexham Maelor Hospital** | 1 psychologist  2 social workers | 6 sessions  6 + 6 sessions |
| **Ysbyty Glan Clwyd** | 2 psychologists  1 social worker | 3 + 3 sessions  10 sessions |
| **Ysbyty Gwynedd** | 1 psychologist  1 social worker | 6 sessions  7 sessions |

**Units in Scotland**

| **Name of renal unit** | **Staff available** | **Number of sessions on renal services** |
| --- | --- | --- |
| **Aberdeen Royal Infirmary** | No renal psychosocial staff available | - |
| Iverness- Raigmore Hospital | No renal psychosocial staff available | - |
| **Dundee- Ninewells** | 1 social worker (shared with oncology) |  |
| **Airdrie – Monklands Hospital** | 2 social workers | 10+ 6 sessions |
| **Glasgow- Yorkhill** | 2 psychologists | 3 + 4 sessions |
| **Glasgow Renal and transplant unit** | No renal psychosocial staff available |  |
| **Dumfries & Galloway Royal infirmary** | No dedicated renal service.  Clinical Health Psychology team has four full time members of staff. Operates a general medical model and this includes renal medicine, but it has no allocated renal time. |  |
| **Crosshouse Hospital** | No renal psychosocial staff available. (General psychology and social work service) | - |
| **Edinburgh Royal Infirmary** | 1 psychiatrist  3 social workers | 4 sessions  10 +6+6 sessions |
| **Kirkcaldy** | No renal psychosocial staff available | - |
